# Targeting the Brain Reservoirs: Toward an HIV Cure

**DOI:** 10.3389/fimmu.2016.00397

**Published:** 2016-09-30

**Authors:** Céline Marban, Faezeh Forouzanfar, Amina Ait-Ammar, Faiza Fahmi, Hala El Mekdad, Fadoua Daouad, Olivier Rohr, Christian Schwartz

**Affiliations:** ^1^INSERM UMR 1121 Faculté de Chirurgie Dentaire, Université de Strasbourg, Strasbourg, France; ^2^EA7292, DHPI, Université de Strasbourg, Strasbourg, France; ^3^IUT Louis Pasteur de Schiltigheim, Université de Strasbourg, Schiltigheim, France; ^4^Institut Universitaire de France, Paris, France

**Keywords:** brain, reservoirs, latency, cure, cART, HIV transcription

## Abstract

One of the top research priorities of the international AIDS society by the action “Towards an HIV Cure” is the purge or the decrease of the pool of all latently infected cells. This strategy is based on reactivation of latently reservoirs (the shock) followed by an intensifying combination antiretroviral therapy (cART) to kill them (the kill). The central nervous system (CNS) has potential latently infected cells, i.e., perivascular macrophages, microglial cells, and astrocytes that will need to be eliminated. However, the CNS has several characteristics that may preclude the achievement of a cure. In this review, we discuss several limitations to the eradication of brain reservoirs and how we could circumvent these limitations by making it efforts in four directions: (i) designing efficient latency-reversal agents for CNS-cell types, (ii) improving cART by targeting HIV transcription, (iii) improving delivery of HIV drugs in the CNS and in the CNS-cell types, and (iv) developing therapeutic immunization. As a prerequisite to these efforts, we also believe that a better comprehension of molecular mechanisms involved in establishment and persistence of HIV latency in brain reservoirs are essential to design new molecules for strategies aiming to achieve a cure for instance the “shock and kill” strategy.

## Introduction

Combination antiretroviral therapy (cART), introduced in 1996, has radically improved the management of HIV-1 infection and decreased both morbidity and mortality. However, despite initial hopes to cure HIV, treatments were unable to fully eliminate the virus (1–3). Indeed, with very sensitive methods (4–6), a remaining viremia is always noticed in patients on cART. Moreover, HIV RNA returns to a measurable plasma level when cART is disrupted (7, 8). The origin of this persistent viremia is still a matter of debate (9–11). Latent persistence of HIV in long-lived cells, such as the central memory CD4+ T-cells, hematopoietic stem cells, dendritic cells, and cells from the monocyte–macrophages lineage in the form of proviruses have been described (1, 2, 12–19). Moreover, these cells are located in a variety of anatomical sites, including tissues, such as blood, brain, gut-associated lymphoid tissue, bone marrow, and genital tract (20), making it difficult to purge the virus from all the reservoirs.

These latently infected cells are from time to time reactivated and produce HIV particles at low levels, thus explaining the persistence of viremia. An alternative theory, the cryptic ongoing replication states that despite cART, HIV is continuously produced at low levels. The inefficiency of the treatment in cells supporting ongoing replication could be due to poor drug penetration in sanctuaries, such as the brain (21) or by cell-to-cell transfer of the virus (22). In theory, there are critical therapeutic implications for cART as it is expected that during ongoing replication, drug resistance might arise (23–26). The potential mechanisms of HIV persistence have been discussed recently in a review by Hong and Mellors (27).

One of the main debates in the field of HIV reservoir is whether or not the central nervous system (CNS) constitutes a real viral reservoir. Indeed, with its unique features, such as the existence of a blood–brain barrier (BBB) with poor drug penetration, the CNS might be considered as a sanctuary (20) made of specific cell types (28) with reduced immune surveillance. Moreover, the anatomy of the CNS is such that there is poor viral genetic information exchange with the other sites and, thus, might be referred as a compartment (20, 29, 30).

First, we will give our opinion on the existence of viral reservoirs in the CNS referring to excellent recent reviews in this topic. Next, we will discuss the importance to purge these potential viral reservoirs. Indeed, in theory, it is possible to acquire virus resistance to cART if there is an ongoing replication in the brain. Another major concern is the existence of HIV-associated neurocognitive disorders (HAND). In up to 50% of the HIV-infected patients on efficient cART and undetectable virus load (≤50 copies/ml), HAND has been recorded. Several mechanisms are evoked to explain the increase of less severe forms of HAND in which production of some viral proteins occurs during reactivation or cryptic ongoing HIV replication. We will then review the state of art of what is known regarding the molecular mechanisms underlying the establishment and persistence of HIV in the potential reservoirs in the brain and, finally, discuss the profound therapeutic implications of purging reservoirs.

## Can the CNS be Qualified as a HIV Reservoir?

A viral reservoir is an infected cell population that allows persistence of replication-competent virus in patients under cART (20). According to this definition, the only true reservoirs are the resting CD4+ T-cells. Indeed, these cells fulfill all the criteria to be considered as a real reservoir, i.e., presence of integrated virus in long-lived cells, persistence of high levels of virus in a quiescent/latent state in the reservoir and possible reactivation of the virus with the formation of replication-competent particles (31).

There are several evidences that brain cells harbor genome-integrated HIV (28). We know that the virus invades the brain very soon following infection. Virus infection was shown in astrocytes (32), in perivascular macrophages (32), and in microglial cells (33). All three cell types are long-lived cells with perivascular macrophages (34) and astrocytes (35) with a half-life ranging from months and microglial cells with a half-life of years (36). All these cells are infected at high frequency in the brain. Astrocytes, the most abundant cell type in the brain, are infected in up to 19% of the cell population (37). Similar ratio of infected cells has been found among the perivascular macrophages and the microglial cells (33, 38). In addition, several mechanisms, including epigenetic regulation, have been evoked to induce latency in these cells notably in astrocytes and microglial cells (39–42).

Due to ethical and technical problems, it is not possible to evaluate the human brain-infected cells for their capacity to produce replication-competent viruses. However, there are several indirect evidences showing that CNS is a reservoir for HIV. Indeed, HIV DNA has been detected in brain tissues isolated from autopsies of HIV patients whose infection has been controlled by cART (33, 39). Moreover, there is a strong correlation of the amount of HIV DNA found in astrocytes and HIV-associated dementia (HAD) (37). Various animal models have been used to show persistence of HIV infection in the CNS as brain biopsy is not possible. Indeed, several animal models, such as macaque, rats, and humanized BLT mouse, have been used to mimic the condition of HIV-infected patients on cART, which confirmed the presence of viral RNA or viral proteins in the brain (43–45). Specifically, in the macaque model, a mechanism of the establishment of transcriptional HIV latency in the CNS has been suggested (46). They notably showed that interferon beta repressed SIV LTR activity by inducing C/EBPγ expression, a dominant negative isoform of C/EBPβ (47). There are also several evidences supporting continuous CNS perturbation despite an efficient cART (48) with an increase of the prevalence of milder form of HAND. Moreover, in patients under suppressive cART activation of the immune system is still observed in the CNS with some biomarkers, such as neopterin or NFL being detected in the cerebrospinal fluid (CSF) (49). One explanation is the existence of an inflammatory process that might be driven by low-level HIV replication in infected cells (50, 51). Interestingly, neuroimaging data are also in favor of persistent CNS inflammation in patients on cART (52, 53). Finally, development of highly sensitive methods, such as single-copy assay (SCA), has allowed the detection of HIV RNA in the CSF from infected patients on cART or from elite controllers whose HIV RNA level was initially undetectable in the plasma and CSF (54–56). The recent discovery of a CSF viral escape in patients on cART with undetectable plasma HIV RNA but with neurological impairment argue also for the existence of a persistent HIV reservoir in the brain (55–59). In conclusion, there are now several evidences supporting that CNS is a reservoir for HIV even if it is still controversial. Readers will be referred to the following reviews that nourish the debate of whether or not CNS serves as a HIV reservoir (60–63).

## Why is it Important to Purge the CNS Reservoir of HIV?

The CNS is involved in the control of most functions of the body and mind. The brain operates in a very well controlled microenvironment separated from the other parts of the body by two barriers: the choroid plexus and the BBB. The two barriers, but predominantly the BBB, constitute physical barriers and any perturbation of their integrity will be associated with neurological diseases. There are several other features that make the CNS unique. The CNS has specific immunological features; principally an innate immune response through the perivascular macrophages and the microglial cells. However, the adaptive immune response has also been observed and, thus, contributes to the immune surveillance in the CNS (64, 65). Indeed, leukocytes trafficking to the CSF either by traversing the BBB to the perivascular space or the choroid plexus has been detected (66). More interestingly, in patients having CSF/plasma HIV discordance (patients having higher levels of HIV RNA in CSF than in blood) even at very low levels it was demonstrated that both innate (macrophages and microglial cells) and adaptive (T CD4+ and CD8+ lymphocytes) are involved in CNS injury (67–70). It has been shown that the percentage of a specific set of T CD8+ lymphocytes that expresses interferon γ is higher in the CSF than in blood. Moreover, this higher percentage of T CD8+ cells in CSF versus blood contributes to the occurrence of HAND (67) [reviewed in Ref. (71)]. Within 2 weeks following acute infection by HIV, the virus enters the CNS. There are at least two mechanisms to explain how HIV crosses the BBB, including trafficking of cell free virus and infected cells (72). The well-documented infection of the CNS is accomplished through infected cells and, thus, has been named the “Trojan Horse” mechanism (73). A recent study using natalizumab, an anti-α4 blocking antibody preventing both lymphocytes and monocytes trafficking across the BBB, is in accordance with this mechanism. Indeed, a drastic decrease of SIV DNA in the brain was observed when natalizumab was given to rhesus macaque during acute SIV infection (74). According to this theory, infected monocytes cross the BBB and infect the perivascular macrophages, the microglial cells, and the astrocytes that result in HIV-associated neurological disorders (75). Since the introduction of cART, an important decrease in the incidence of the severe form of HAND has been noticed (76). However, there is an increase of milder form of the infection (up to 50%), which might be largely under diagnosed. Thus, better screening tools to detect HAND are required in the future (77). The reasons for the increase of the prevalence of milder forms of HAND are not fully understood. One explanation might be related to the existence of quiescent/latent viral reservoirs in the CNS that emphasizes the importance of eradicating the reservoirs. Another major concern related to the existence of such quiescent/latent reservoirs in the CNS is that it might be a source of new particles that could replenish the periphery blood. These notions will be discussed in the later chapters.

### HIV-1 and HIV-Associated Neurological Disorders

HIV-associated neurocognitive disorders have been divided into three subgroups according to the Frascati criteria, i.e., asymptomatic neurocognitive impairment (ANI), mild neurocognitive disorder (MND), and HAD (78). These disorders are associated with the entry of HIV into the CNS that occurs almost immediately after systemic infection (79). The more severe form of HAND, i.e., HAD has drastically decreased with the introduction of cART. However, the less severe forms (MND and ANI) have continued with a prevalence ranging from 20% to up to 50%, while keeping in mind that these milder forms are often under diagnosed (80, 81). However, the details of persistence of these less severe forms of HAND in patients on cART are not fully understood. There are at least two hallmarks of HIV infection in the brain, i.e., chronic immune activation and compromised BBB integrity in which the central role for HIV neuropathogenesis is played by the monocytes/macrophages (82–85). Importantly, immune activation still occurs in patients on cART (50, 51). The exact mechanisms of such pathogenesis are not entirely known and rely on two models: a direct and an indirect model (86, 87). In the direct model, infected cells will cause neuronal death through the action of newly synthesized viral proteins, such as Tat, gp120, Vpr, and Nef. The two major viral proteins that lead to neuronal injury are Tat and gp120. Their effects are mediated through their interaction with neuronal cell receptors, such as the NMDA receptor and the chemokine receptors (CCR5 and CXCR4). More details on the mechanisms involved in the neuropathogenesis caused by viral proteins are found in the review (88). In the indirect model, sustained chronic inflammation is induced by secreting perivascular macrophages, microglial cells, and to a lesser extent by astrocytes releasing neurotoxic host factors. Among these secreted products, there are proinflamatory cytokines (TNFα, IL-1β, IL-6, IL-8, and INFα), chemokines (CCL2 and CCL5), and small molecules, such as quinolinic acid and the platelet-activating factor. Moreover, these viral proteins and cellular factors increase the oxidative stress and alter the integrity of the BBB which in turn results in the stimulation of even more infected cells in the brain. Further investigations are needed to decipher the exact mechanisms involved in CNS injury. Interestingly, Tat might be involved in both direct and indirect processes that lead ultimately to neuronal death. Potential roles and functions of Tat in both direct and indirect neurotoxicities have been described elsewhere (89, 90). The importance of Tat is still a matter of debate since there are controversies regarding the amount of Tat present in the CNS cells environment and the amount of Tat used in *in vitro* experiments. In favor of its importance is the use of Tat transgenic animal model where CNS injury has been observed (91, 92). Therefore, it will be essential to detect Tat in the brain from patients on cART. It is possible that this protein might arise from quiescent/latent reservoirs and, therefore, be responsible for the milder form of HAND. Improvement of cART by targeting the production phase of HIV-1, including transcription appears, therefore, crucial (93). Indeed, current cART is not targeting this step and since the CNS infection occurs almost immediately during acute infection, establishment of infected reservoirs will not be prevented. Moreover, strategies aiming to purge the reservoirs are based on HIV reactivation with the risk that viral proteins, such as Tat will be produced in the brain. HIV-1-mediated neuropathogenesis might also involve a dynamic interaction between astrocytes and peripheral blood mononuclear cells (PBMCs) (94). Indeed, a recent report showed that astrocytes susceptibility to produce HIV infection is enhanced by PBMCs producing interferon γ which in turn inhibit HIV-1 production in PBMCs through the secretion of small glycoprotein, i.e., the Wtns. These later proteins have been shown to be involved in many CNS processes (95), such as synaptic plasticity and neurotransmitter release, which might explain partly HIV-1-mediated neuropathogenesis.

### CNS Reservoirs as a Source of Virus

The CNS has two special features making it difficult the achievement of a cure. First of all, the CNS is considered as a sanctuary for HIV by pharmacologic means as it is a site with limited access to antiretroviral drugs (ARV) (96–99). As an outcome, there is a risk to allow the occurrence of virus resistant to the current drugs used in cART. Second, the CNS is also considered as a compartment in which the virus is isolated from other parts of the body (29, 100). Because of poor genetic information exchange with the other sites, neurotropic variants of HIV might be selected, which most likely will not respond to treatment in a similar way than the virus encountered in the CD4+ T-cells, the main target in the body. There are now numerous evidences supporting the fact that the CNS-resident virus has evolved to become macrophage tropic (101). Indeed, sequence analysis of the *env* gene and of the HIV-1 promoter (LTR) argue for the compartmentalization of HIV variants in the CNS (102–105). Variations in the promoter are important since mechanisms involved in the establishment and persistence of latency in the CNS might differ from the one described in CD4+ T-cells. As mentioned above, this will impact the efficiency of latency-reversing agents (LRA) in strategies aiming to purge the latent/quiescent reservoirs (106, 107). Another major concern regarding the necessity to purge the CNS reservoirs is related to the discovery of CNS viral escape in patients on cART (108). Initial studies have shown occasional cases of virus escapes in the CSF (109, 110). Development of highly sensitive assays has even allowed the detection of CSF HIV RNA, which were not detectable with previous assays (111). Indeed in a report, evaluation of CSF viral escape has been done in a cohort of neurologically asymptomatic patients successfully treated with cART. It was shown that around 10% of these patients had detectable CSF HIV RNA, suggesting that viral escape may be underestimated (112). The recent discovery of a CNS viral escape in a cohort of 14 patients on cART with undetectable plasma HIV RNA but who developed HIV-encephalitis argues for the possibility that CNS is a real reservoir (57). Actually, this study and others raise the question that CNS-specific viral replication can occur in patients on cART from reactivated reservoirs which in theory may have escaped therapy and ultimately lead to drugs resistance (58, 59, 113). Very interestingly a similar drug-privileged site, i.e., the lymphoid tissue has been shown to have low access to drugs (114). The authors notably showed that the virus is continuously produced and might be a source of HIV from which replenishment of blood occurs. However, and contrary to the brain, they do not show that resistance to antiretroviral drugs arises. The authors of this study suggest that this absence of resistance to ARV might be explained by the too low level of drug concentration in lymphoid tissue that is not sufficient to confer competitive advantages to the development of drug-resistant viruses. This study point out to the importance of developing new ways to deliver drugs in all sanctuaries, including brain and lymphoid tissues (115).

Overall, we suggest that it is crucial to eradicate brain reservoirs since ARV-resistant viruses are capable to replenish the systemic circulation from these reservoirs. It will also imply that CSF analysis in patients on cART should be performed more often since it will greatly help assessing the compartmentalization of HIV in the brain and monitoring the efficiency of new treatments (116). Notably, CSF might be used to evaluate HIV drug resistance.

## Molecular Mechanisms of HIV-1 Latency

Establishment and persistence of HIV latency occur in brain cells, i.e., perivascular macrophages, microglial cells, and astrocytes. Infection of these cells differs from the infection of blood cells infected, mainly the CD4+ T-cells. Indeed, HIV infection in macrophages is not lytic and these cells are far more resistant to cytopathic effects. Moreover, infected monocyte–macrophage cells are also more resistant to apoptosis, a major obstacle for the eradication of the virus. These cells may harbor latent viruses for months (perivascular macrophages) or for years (microglial cells). Astrocytes are also thought to be infected by HIV-1 despite the lack of the co-receptors CCR5 and CXCR4 probably through the involvement of vesicles (38). However, the infection appears to be non-productive with only early transcripts, such as *tat* and *nef*, that are detectable at very low level (117).

**Figure 1:**
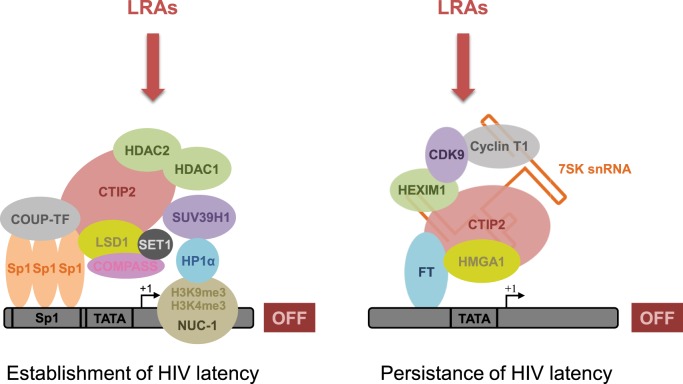
**CTIP2 promotes the establishment and persistence of HIV-1 latency through the recruitment of two macromolecular complexes on the HIV-1 promoter in microglial cells**. CTIP2 participates in the establishment of HIV-1 latency by recruiting a chromatin-modifying complex at the HIV-1 promoter. This complex consists of two histone deacetylases: HDAC1 and HDAC2 that are responsible for H3K9 deacetylation of Nuc-1, a nucleosome located immediately downstream of the HIV-1 transcriptional start site. The histone methyltransferase SUV39H1 takes also part of the complex and catalyzes the tri-methylation of H3K9 on Nuc-1. Finally, HP1α, a protein associated with heterochromatin, specifically recognizes H3K9me3 and spreads along the HIV-1 promoter, thus creating a domain of heterochromatin refractory to transcription. In parallel, CTIP2 also recruits the histone demethylase complex LSD1/COMPASS/SET1 that, in association with the histone marks H3K9me3 and H3K4me3, contributes to HIV-1 gene silencing and, therefore, the establishment of HIV-1 latency. Besides, by recruiting a transcriptional inhibitory complex at the HIV-1 promoter, CTIP2 is also involved in the prevention of HIV-1 reactivation. This complex is an inactive form of the elongation factor pTEFb and consists of pTEFb, HEXIM1, HMGA1, and the snRNA 7SK. Due to their involvement in HIV-1 establishment and persistence of HIV-1 latency, CTIP2-associated proteins from both complexes constitute new pharmacological targets to reverse HIV-1 latency. Accordingly, new latency-reversing agents (LRAs) are currently being developed or undergoing clinical trials with the aim of reversing HIV-1 latency and depleting HIV-1 reservoirs.

Understanding the intimate mechanisms underlying HIV-1 latency in these CNS-specific cells is necessary to develop new and original therapies for viral eradication. The molecular mechanisms underlying these therapies are determined by the cellular specificity of HIV gene transcription and the variability of the LTR found in viruses having evolved in the brain (61, 118). For example, it has been shown in microglial cells that Sp3 and a truncated form of C/EBPβ (NF-IL6) inhibit the basal transcriptional activity of HIV-1 (47). Such a reduced basal and Tat-activated transcriptional activity has also been shown in astrocytes. Transcriptional silencing has been associated with low levels of TAR RNA binding proteins (TRBP) and with mutations of the SP motifs found within the LTR of brain-derived HIV-1. Mutations prevent the transcription factor Sp1 to bind the promoter and, thus, inhibit transcriptional activation (119, 120). However, the main mechanism involved in establishment and persistence of latency involves epigenetic regulation (41, 121, 122). In our laboratory, we showed that the cellular factor COUP-TF interacting protein (CTIP2) is a key factor in the establishment and persistence of HIV latency in microglial cells (123). We notably showed that this protein serves as a platform to anchor several protein complexes having different functions. Indeed, at least two different complexes containing CTIP2 are involved in the establishment and the persistence of HIV-1 latency (Figure 1). Moreover, CTIP2 is also involved in the control of cellular genes of importance for the virus. Among these factors, the cellular cyclin-dependent kinase inhibitor CDKN1A/p21^waf^ has been described to favor HIV-1 gene transcription in the monocyte–macrophage lineage. This effect indirectly favors HIV-1 latency since activation of the p21 gene stimulates viral expression in macrophages (124). Moreover, CTIP2 counteracts HIV-1 Vpr protein that is required for p21 expression (125). We, therefore, suggested that CTIP2 generates a cellular environment disfavoring viral reactivation and, thus, favoring HIV-1 latency.

The first CTIP2-associated complex described in our laboratory has been involved in the establishment of HIV-1 latency through the induction of heterochromatin in the vicinity of the viral promoter (Figure 1, left complex). Indeed, we showed that CTIP2 recruits a chromatin-modifying complex through the Sp1 sites of the proximal promoter (42). This complex contains the histone deacetylases HDAC1, HDAC2, and histone methyltransferase SUV39H1 that specifically demethylates lysine 9 of histone H3. This histone modification allows heterochromatin protein 1 (HP1) binding, heterochromatin formation, and hence HIV silencing (42, 126, 127). In a consecutive study, we have shown that CTIP2 interacts physically and functionally with the lysine-specific demethylase (LSD1) to repress HIV-1 transcription and viral expression in a synergistic manner (128). The recruitment of LSD1 at the HIV-1 proximal promoter has been associated with both H3K4me3 and H3K9me3 epigenetic marks, which is linked to the recruitment of hSet1 and WDR5, two members of the hCOMPASS complex, on the HIV-1 promoter (128). Recruitment of CTIP2 on the p21 promoter also induces a heterochromatin environment. Moreover, CTIP2 has been shown to silence p21 gene transcription by creating epigenetic marks of repression, as described above for the HIV-1 promoter (125). Interestingly epigenetic regulation of HIV-1 latency, which was associated with the recruitment of HDACs and SUV39H1 has also been described in astrocytes (40). Finally, in a recent report, investigation of the neuropathology and the molecular alterations associated with CNS latent HIV-1 infections provided evidence that HIV-1 persistence in the brain is associated with high level of CTIP2, HDACs, and HP1 (39).

We also showed that CTIP2 belongs to another complex able to prevent HIV-1 reactivation (Figure 1, right complex) (129). Indeed previous work has shown that CTIP2 represses the late phase, Tat-dependent, of HIV-1 transcription (127). In the absence of the trans-activator factor Tat, an inactive form of the elongation factor pTEFb is found in a multiprotein complex, including 7SK snRNA, CTIP2, and HEXIM1 anchored to viral and cellular gene promoters (129). pTEFb is composed of a regulatory subunit CyclinT1 and a catalytic subunit CDK9, whose kinase activity is involved in the Ser2 phosphorylation of the carboxyl terminal end of the RNA polymerase II and in the phosphorylation of the negative transcriptional elongation factors NELF and DSIF. Following phosphorylation, the RNA pol II processivity significantly increases, which leads to an efficient transcription of genes (130). Interestingly, we have shown that CTIP2 drastically repressed CDK9 kinase activity in this inactive complex, thus, inhibited pTEFb function. Finally, we showed that the cellular protein high mobility group AT-hook 1 (HMGA1), which also belongs to the 7SK snRNA complex recruits the inactive CTIP2/pTEFb complex to the HIV-1 and cellular target promoters (131). As a consequence, protein complexes containing CTIP2 regulate viral and endogenous gene expression, thus favoring HIV-1 persistence. Far more investigations are still needed to decipher the precise molecular mechanisms involved in these processes. We still do not fully understand how the transition from transcription initiation into elongation (which involves pTEFb) is controlled by cellular factors and/or the viral transactivator Tat. We and others hypothesized that the inactive form of the pTEFb complex is part of a 7SK complex that is anchored to the promoter by either CTIP2 (129) or Kap1 (132), thus available for RNApolII elongation through its activation. The transition from the inactive to the active form of the pTEFb complex through the action of Tat is not well understood but may involve a phosphatase (PPM1G/PP2Cγ) that takes apart pTEFb from the 7SK complex (133).

## Therapeutic Implications for the Eradication of HIV-1 from Brain Reservoirs

Several considerations already mentioned [emergence of multidrug resistance (24, 113), non-AIDS-related events (134–136) etc.] urge the search of new ways to develop a sterilizing or a functional cure for AIDS (137). The purge of viral reservoirs by the “shock and kill” strategy (138) is a possible approach to achieve such a cure. This strategy aims at purging or at least reducing the size of cellular reservoirs by reactivating HIV transcription (shock) followed by intensive cART therapy and immune activation (kill) (139, 140). As several reports suggested, using LRA alone or in combination have proven the efficiency of this strategy in the reactivation of quiescent/latent HIV from CD4+ T-cells reservoirs (138, 141–145). Several clinical trials have been carried out and some others are in progress or forthcoming (146). This strategy of reactivation needs to work on all potential reservoirs, including brain reservoirs. However, several limitations to the eradication of the brain reservoirs may preclude a cure.

### Limitations to the Eradication of Brain Reservoirs

It is essential to decipher the molecular mechanisms underlying HIV persistence in all types of potential reservoirs, since some important differences in those mechanisms have been noticed in all latently infected cell types. For example, LSD1 has been associated with activation of HIV transcription in CD4+ T-cells (147). However, in microglial cells, LSD1 played a role in the establishment of latency (128). LSD1 mediates HIV-1 transcription silencing in microglial by anchoring various factors at the promoter rather than inducing HIV-1 transcription by its own enzymatic activity in CD4+ T-cells. The dual role of LSD1 achieved by different mechanisms in the two main HIV-1 cellular targets points to the complexity of the molecular mechanisms of HIV latency (148). Hence, additional investigations of the epigenetic regulation of HIV latency are needed in order to develop efficient drugs targeting each potential viral reservoir. Furthermore, as mentioned in the previous sections, there are several characteristics of the CNS, which limits a cure by the “shock and kill” strategy:

- The CNS is a sanctuary with barriers (BBB and choroid plexus) that reduce the access of some of the drugs currently used to the brain (97).
- The main cellular targets are astrocytes and CNS-resident macrophages. However, few drugs are able to target the monocyte–macrophages lineage (149) and the effects of cART on HIV replication in astrocytes are unknown or neurotoxic (150).
- CNS has long been considered as an immunologically privileged site (151). Therefore, achieving immune activation through cytotoxic T lymphocytes (CTL) activation to eliminate the potential reservoirs may be difficult or even deleterious in the brain.
- Another major concern is related to the fact that reactivation of the virus with LRA will lead to the synthesis of neurotoxic viral proteins, such as Tat and the gp120, as there are no drugs currently available targeting HIV transcription. Moreover, reactivation of the virus is often associated with CNS inflammation through macrophage/microglial cell activation (152, 153).

### How Can We Overcome These Limitations?

With these limitations evoked in the previous section, it may be difficult to achieve a purge in the CNS. The idea is to eliminate or reduce the pool of latent/quiescent reservoirs with the aim to mimic elite controllers able to control the HIV infection and with very low amount of reservoirs. Introducing cART very early following HIV infection has been proved to be efficient since it limits the size of the latent/quiescent reservoirs (154–156).

In our opinion, achieving a sterilizing cure or a partial functional cure in the brain needs efforts in four directions: (i) designing efficient LRA for CNS-cell types, (ii) improving cART by targeting HIV transcription, (iii) improving delivery of HIV drugs in the CNS and in the CNS-cell types, and (iv) developing therapeutic immunization.

Designing potent LRAs to reactivate HIV-1 transcription from the CNS-cell types is crucial in a “shock and kill” strategy. However, we and others have shown that the molecular mechanisms involved in the establishment and persistence of latency in these cells may differ from the mechanisms involved in the CD4+ T-cells that are currently the main targets for LRAs (106, 107, 137). As a consequence, the outcome in the use of LRAs may differ in CNS-cell types. Several HDAC inhibitors (HDACi) have been tested in the U1 monocyte cell line and in primary cells (astrocytes and macrophages) (106, 107, 157). Preliminary data showed that some LRAs, including panobinostat (158) and JQ1 (159), are relatively non-toxic and efficient to induce HIV reactivation at a therapeutic concentration (106, 107). On the contrary, other LRAs, including disulfiram and vorinostat, which were promising in CD4+ T-cells, were not working at therapeutic concentration in the CNS-cell types (106, 107). Among LRAs, bryostatin-1 is very promising since it can cross the BBB to activate brain Protein Kinase C especially in the two main targets for HIV-1, i.e., microglial cells and astrocytes (142, 160). This PKC activator has already been used in both preclinical trials for Alzheimer disease and in clinical trials to treat cancers [reviewed in Ref. (161)]. Further investigations will be needed to characterize new targets, such as the hCompass complex, recruited on the viral promoter by LSD1 in microglial cells. Preclinical studies in animal models are also needed to test the efficacy of LRAs. Combinations of LRAs have to be tested *in vitro* and *in vivo* as well, since they may work in a synergistic manner as described (142, 162). Using combination of LRAs with lower dose may also prevent some drug side effects when used alone at a higher concentration [reviewed in Ref. (163)]. Finally, a recent pilot study has suggested that administration of panobinostat, a potent activator of HIV transcription in CNS-cell types, was not associated with side effect in the brain as assessed by CSF biomarkers, such as neopterin, C reactive protein, and IP-10 (164).

Improving cART by targeting HIV transcription is also crucial since there are currently no drugs targeting this step (93). Moreover, reactivation of HIV leads to the synthesis of neurotoxic viral proteins, such as Tat. We and others discussed in details the importance of targeting this step and readers are referred to these recent reviews (93, 165). Particularly, inhibitors may be developed against the two main targets that control HIV transcription, i.e., the cellular factor NF-KB and the viral transactivator Tat. Since NF-KB also plays a central role in inflammation, new drugs targeting this factor will also prevent or at least reduce chronic inflammation in the brain (166, 167). It is also important to target the viral transactivator Tat since this factor is involved in the regulation of HIV-1 and its secreted form induces neuronal death by direct neurotoxicity. Several molecules, especially natural compounds deserve attention (168, 169). We believe that characterization of new targets associated with the exploitation of new technologies, such as bioengineering, high-throughput screening, computer-aided drug design, and combinatorial chemistry, will considerably improve the discovery of new drugs. Among the molecules that deserve attention, we can mention the dCA, a chemical derivative of corticostatin, a natural steroidal alkaloid isolated from a sponge (170–172). A promising Tat inhibitor has been recently isolated from the plant *Tripterygium wilfordii* and named triptolide (173). This molecule, which is currently in phase III of a clinical trial, inhibits both HIV replication and transcription by increasing the proteasomal degradation of Tat. Another family of protein that deserves attention is the DING proteins (pDINGs), a family of potential therapeutic agents against HIV-1 (174–178). These molecules discovered in bacteria, plants, and animals have been reported to inhibit HIV transcription. In addition, it has been shown that a phosphorylated form of pDING is a neuroprotective factor and could be used to reduce neuro inflammation due to HIV-1 (88, 179).

Another major limitation to purge brain reservoirs is related to the poor access of the drugs in the CNS due to the presence of barriers, such as the BBB. Moreover, drugs have to target macrophages and astrocytes. Indeed, it has been shown that all drugs, except protease inhibitors, display reduced activity in macrophages compared to CD4+ T-cells (180). We have already mentioned that some LRAs have no effect in the CNS-cell types at a therapeutic concentration. Different mechanisms have been evoked to explain the lower EC50 values of these drugs in macrophages/microglial cells. Drug penetration may be reduced due to the differential expression of efflux transporter and multidrug resistance proteins (181, 182). Several ways are explored to overcome these limitations and discussed in other reviews (183, 184). Improvement of both bioavailability and bio-distribution of drugs used in cART will increase the access of these drugs to the brain. Among the approaches used to improve drug delivery in the brain, there is the development of carriers, such as liposomes, dendrimers, and micelles. A particularly promising approach is based on polymeric nanomedicines that raise hope for eradication of HIV from all potential reservoirs [reviewed in Ref. (185–187)]. Increase in treatment efficacy and tolerance may be expected, hence favoring patient adherence. These later strategies may also increase the distribution of drugs in CNS-cell types, such as astrocytes and macrophages/microglial cells. Indeed, macrophages/microglial cells constitute an important but neglected barrier for HIV eradication, which will need efforts to circumvent (149, 188). Several conventional and new therapeutics against HIV-1 in macrophages, including PI3K/Akt blocking agents, carbohydrate-binding agents, and small interfering RNAs, have been discussed elsewhere and deserve real attention (184, 189).

Immune-based therapeutics should also be considered since the size of the reservoir following treatment with LRAs is not reduced and need immune activation to clear them (190). In particular, CTL activation has been shown to clear HIV-1 from infected CD4+ T-cells (140). Previous studies done in animal models argued in favor of the importance of CTL in the clearance of HIV-1 infected macrophages in the brain (191, 192). A CD8+ T-cell response appears essential in the control of other brain infections, such as toxoplasmosis (193). Dealing with immune activation is not easy and constitutes a challenge for strategies aiming to eradicate HIV-1 reservoirs. These approaches need further investigations and development of adequate animal models to ensure the feasibility of such treatments (194, 195). Another unexplored non-conventional way to clear reactivated latently infected cells is based on the use of neutralizing antibodies against HIV-1 with promising results obtained in humanized mice (143, 196).

## Conclusion

Reducing the size of reservoirs is fundamental for HIV+ patients to control their viral replication without any treatment, a situation typical for elite controllers. The purge of HIV reservoirs constitutes, therefore, one of the top research priority of the International AIDS Society (IAS) through the action “Toward an HIV Cure.” We may expect to get a sterilizing cure by eradicating the virus from all the reservoirs but a more realistic view would be a functional cure through the reduction of the pool of cellular reservoirs. A major problem is to reduce/eradicate reservoirs located in the CNS. There are now numerous direct and indirect arguments for the existence of a pool of quiescent/latent reservoirs in the brain even if it has not been demonstrated in human yet. The strategy called “shock and kill” enables reactivation of quiescent/latent reservoirs followed by an intensive cART to clear the reservoirs. Several pilot clinical trials have been done and some are ongoing and upcoming. The results of trials are encouraging but also point to the need of additional interventions, such as immune activation, in order to clear the reservoirs. This immune activation approach is needed to eliminate or reduce brain reservoirs but might be difficult since the CNS has several unique characteristics. Indeed, the CNS is a pharmacological sanctuary and a compartment isolated from the other parts of the body. In addition, latently infected cells in the brain, i.e., astrocytes and macrophages/microglial cells are rather different from the main memory T-cells reservoir. Altogether, intense efforts are needed in several directions, including the design of efficient LRAs for CNS-cell types, improving cART by targeting HIV transcription, improving delivery of HIV drugs in the CNS and in the CNS-cell types and developing therapeutic immunization therapies in order to overcome the above discussed limitations. We believe that we are at a crossroads to achieve a cure for HIV. Indeed, there are several adequate animal models (non-human primate, humanized mice, etc.) to test the efficiency of strategies aiming to purge reservoirs. Identification of new targets and the availability of new technologies will also allow the design of new original drugs. In particular, new natural compounds and their derivatives could help in the design of new class of molecules targeting HIV-1 transcription a step not yet targeted by cART. This is especially crucial in a strategy aiming to reactivate latent CNS-cell types. Finally, hope rises also with the advent of nanotechnologies. Although still in the early stage, nanotechnologies could be used in drug transport to enable drugs to reach both the brain (by crossing barriers such as the BBB) and the CNS-cell types (by crossing cell membranes). Dosage is expected lower and in consequence less toxicity and a better adherence to treatment is awaited.

## Author Contributions

CM revised the manuscript and made substantial contributions to its final content and design. AA-A, FFo, FFa, FD, and HM revised the manuscript. OR and CS drafted the manuscript. All authors read and approved the final manuscript.

## Conflict of Interest Statement

The authors declare that the research was conducted in the absence of any commercial or financial relationships that could be construed as a potential conflict of interest.
